# Primary ciliary dyskinesia: critical evaluation of clinical symptoms and diagnosis in patients with normal and abnormal ultrastructure

**DOI:** 10.1186/1750-1172-9-11

**Published:** 2014-01-22

**Authors:** Mieke Boon, Anne Smits, Harry Cuppens, Martine Jaspers, Marijke Proesmans, Lieven J Dupont, Francois L Vermeulen, Sabine Van Daele, Anne Malfroot, Veronique Godding, Mark Jorissen, Kris De Boeck

**Affiliations:** 1 Department of Pediatrics, Pediatric Pulmonology, University Hospital Leuven, Leuven, Belgium; 2 Center for Human Genetics, KUL, Leuven, Belgium; 3 Department of Otorhinolaryngology, Head and Neck Surgery, University Hospital Leuven, Leuven, Belgium; 4 Department of Pulmonology, University Hospital Leuven, Leuven, Belgium; 5 Department of Pediatrics, Pediatric Pulmonology, University Hospital Ghent, Ghent, Belgium; 6 Department of Pediatrics, Pediatric Pulmonology, Universitair Ziekenhuis Brussel UZB, Brussel, Belgium; 7 Department of Pediatrics, Pneumologie Pédiatrique et Mucoviscidose, UCL St-Luc, Brussels, Belgium

**Keywords:** Primary cell culture, Ultrastructure, Population characteristics, Primary ciliary dyskinesia, Transmission electron microscopy, *DNAH11*

## Abstract

**Background:**

Primary ciliary dyskinesia (PCD) is a rare disorder with variable disease progression. To date, mutations in more than 20 different genes have been found. At present, PCD subtypes are described according to the ultrastructural defect on transmission electron microscopy (TEM) of the motile cilia. PCD with normal ultrastructure (NU) is rarely reported because it requires additional testing. Biallelic mutations in *DNAH11* have been described as one cause of PCD with NU.

The aim of our study was to describe the clinical characteristics of a large population of patients with PCD, in relation to the ultrastructural defect. Additionally, we aimed to demonstrate the need for biopsy and cell culture to reliably diagnose PCD, especially the NU subtype.

**Methods:**

We retrospectively analyzed data from 206 patients with PCD. We compared the clinical characteristics, lung function, microbiology and imaging results of 68 patients with PCD and NU to those of 90 patients with dynein deficiencies and 41 patients with central pair abnormalities. In addition, we aimed to demonstrate the robustness of the diagnosis of the NU subtype in cell culture by data from genetic analysis.

**Results:**

PCD with NU comprised 33% (68/206) of all patients with PCD. Compared to other subtypes, patients with PCD and NU had a similar frequency of upper and lower respiratory tract problems, as well as similar lung function and imaging. With the currently widely applied approach, without cell culture, the diagnosis would have been missed in 16% (11/68) of patients with NU. Genetic analysis was performed in 29/68 patients with PCD and NU, and biallelic mutations were found in 79% (23/29) of tested patients.

**Conclusions:**

We reported on the clinical characteristics of a large population of patients with PCD and NU. We have shown that systematic performance of biopsy and cell culture increases sensitivity to detect PCD, especially the subtype with NU.

PCD with NU has similar clinical characteristics as other PCD types and requires biopsy plus ciliogenesis in culture for optimal diagnostic yield.

## Background

Primary ciliary dyskinesia (PCD) is a rare disease [1], caused by congenital abnormalities in both structure and function of the motile cilia. It is characterized by upper and lower respiratory tract infections, an increased incidence of situs inversus (SI) and male infertility. It is a genetically heterogeneous and predominantly autosomal recessive disorder.

To date, mutations causing PCD have been described in more than 20 genes. Specific genes are linked to distinct ciliary ultrastructural abnormalities on transmission electron microscopy (TEM): mutations in *DNAI1, DNAH5, TXNDC3, DNAI2, DNAL1, CCDC114* and *ARMC4* cause PCD with outer dynein arm (ODA) deficiency [2-4], whereas mutations in *DNAAF1, DNAAF2, DNAAF3, HEATR2, LRRC6, CCDC103*, *DYX1C1*, *ZMYND10*, *SPAG1* and *C21orf59* cause combined inner dynein arm (IDA) and ODA deficiency [2,5-8]. Absence or displacement of the central microtubular pair (*RSPH9, RSPH4A, RSPH1, CCDC39, CCDC40*) or absence of the nexin links (*CCDC164*) have also been linked to specific genetic defects [2,9]. *DNAH11* and recently also *HYDIN* and *CCDC65* mutations are reported in some patients with PCD and normal ultrastructure (NU) [10-12]. The reported prevalence of PCD with NU ranges from 10% to just over 20% of all PCD cases [13,14]. However, it has previously been shown to be up to 28% in our lab [15]. Nevertheless, *DNAH11* mutations are rare in population studies [16] and the frequency of PCD with NU remains under discussion.

The diagnosis of PCD is challenging and cannot rely on signs and symptoms alone [17]. Absence of the ODA and/or IDA or abnormalities of the central and peripheral microtubules can be detected by TEM. This technique was once proposed as the gold standard for diagnosis, but will obviously miss PCD with NU. In PCD with NU, only *in vitro* functional evaluation of the ciliary motility can identify absent, diminished or uncoordinated movement. However, secondary ciliary dyskinesia (SCD), defined as a non-inherited dysfunction of the cilia due to respiratory infection or exposure to cigarette smoke, can complicate functional evaluation. SCD should be excluded by repeated biopsy or by cell culture [18]. Because *de novo* regrowth of cilia is induced, SCD can be excluded reliably, increasing specificity to diagnose PCD. Nasal nitric oxide measurements are useful as a screening test for PCD. They are low in most patients with PCD, including those with normal ultrastructure. However, it is not widely available and standardization is lacking.

The phenotypic features of children and adults with PCD have been described previously [19,20], and attempts have been made to detect a correlation between the ultrastructural abnormality and clinical signs and symptoms [21-26]. However, only few data are available on the characteristics of PCD with NU and little is known about the clinical characteristics of these patients in comparison with those of patients with ‘classic’ PCD [11,12,16].

The main aim of our study was to describe clinical characteristics of a large population of patients with PCD, in relation to their ultrastructural defect. We also aimed to demonstrate the need for biopsy and in vitro ciliogenesis to confidently diagnose PCD, especially in the NU subtype. Therefore, we retrospectively compared the results from functional and structural evaluations of the cilia before and after cell culture. Genetic analysis was performed to support the diagnosis of PCD in the subgroup of patients with NU.

## Material and methods

We first described the clinical characteristics of patients diagnosed with PCD, confirmed in the KU Leuven Laboratory of Ciliary Function from January 1990 to August 2012, with special focus on patients with the NU subtype. Additionally, we described the laboratory findings before and after cell culture. The study was approved by the Ethical Committee of the University Hospital of Leuven.

The KU Leuven Laboratory of Ciliary Function is the only facility for in-depth evaluation of ciliary structure and function by cell culture in Belgium. Patients were referred from other centers. Biopsy samples were sent for diagnostic evaluation of PCD. Screening tests such as nasal NO measurements were performed in local patients, but not in all patients referred from other centers because of lack of equipment in some referring centers.

### Assessment of clinical characteristics in patients with PCD

A standardized data sheet was used for data collection including age at diagnosis (by nasal biopsy), gender, ethnicity, parental consanguinity, presence of a sibling with PCD and situs inversus including incomplete situs abnormalities. Data were retrieved retrospectively from the last available clinical records or by contacting patients or treating physicians.

The following signs and symptoms were evaluated as lifetime prevalence (‘has ever had’): neonatal respiratory problems (upper or lower respiratory tract symptoms within the first 2 weeks of life), wheezing, lobectomy, chronic sinusitis (nasal discharge during at least 8 weeks or at least 4 episodes of acute rhinosinusitis each persisting for ≥10 days, or abnormalities on sinus CT scan after 4 weeks of treatment), nasal polyps, sinus surgery, ear discharge (purulent discharge via the ear canal), ear drum perforation, hearing loss (conductive hearing loss ≥20 dB), hearing aid, grommets insertion and adenotomy. Presence or absence of pulmonary infiltrates, lobar consolidation/atelectasis and bronchiectasis were scored on all available chest radiographs and/or CT scans. All respiratory samples (sputum, bronchoalveolar lavage or cough swab), available since diagnosis were evaluated for the presence of *H. influenzae*, *S. pneumoniae*, *S. aureus* and/or *P. aeruginosa*. Lifetime prevalence was reported as ‘has ever had infection with’. Chronic colonization was defined as persistence of the same pathogen in at least 3 sputum samples over a period of at least 6 months. Nasal nitric oxide (nNO) values were reported if known.

The ‘evaluation at last follow-up’ included age, weight, height, BMI (body mass index) and lung function. Weight and height were expressed as z-scores, according to the Flemish growth curves [27]. BMI was calculated as weight (kilograms)/height^2^ (meters) and also expressed as z-scores, according to the Flemish growth curves. Spirometry results were expressed as z-scores according to the reference values of Quanjer and Stanojevic [28]. Symptoms scored at ‘current evaluation’ were: chronic cough (coughing >3 days/week), chronic sputum production (expectorating sputum >3 days/week), clubbing and nasal secretions.

### Diagnosis of PCD by cell culture, TEM and genetics

**Figure 1:**
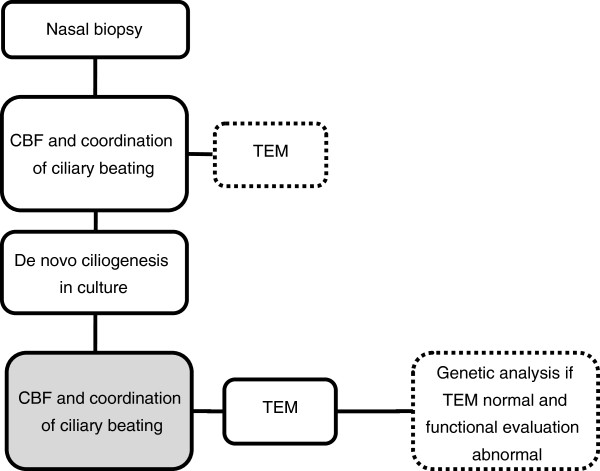
**Flow chart of the diagnostic algorithm for PCD, used in our center.** Functional evaluation (CBF and coordination of ciliary beating) was performed on the fresh biopsy, provided that cilia were present. If sufficient material was obtained, a part of the biopsy is prepared for TEM, the rest is brought in a cell culture system. After ciliogenesis in culture, all the samples are evaluated for functional and structural abnormalities. The diagnosis of PCD is made when coordination after ciliogenesis in culture (marked in grey) is abnormal. Genetic analysis is not performed routinely, but is performed preferentially in patients with normal ultrastructure.

The flow chart for the diagnostic algorithm of PCD in our center is shown in Figure 1. All patients included underwent at least one nasal mucosal biopsy for functional and structural evaluation of the respiratory cilia before and after ciliogenesis in culture, even if the evaluation before ciliogenesis was normal. In case insufficient material was available for both evaluations, all material was brought into culture.

Nasal epithelial cells were enzymatically dissociated from nasal biopsy specimens. If cilia were seen, ciliary beat frequency (CBF) and coordination of ciliary activity were evaluated. Ciliary movement was recorded with digital high-speed video imaging using a MotionScope high-speed camera mounted on an inverted Olympus microscope. CBF was calculated with Matlab software (version 6.5) (reference values obtained in our laboratory were CBF 7.9 Hz, SD 1.8 at 23°C). Coordination of ciliary motion was evaluated by directly observing movement of fluid and debris in the medium and rotation or migration of aggregates.

If sufficient material was present, TEM was performed on the initial biopsy sample in order to identify any structural abnormalities. Cross-sections were made in the ciliary shaft, neither towards the base nor towards the tip of the cilium. Per sample at least 50 transversal cross-sections were evaluated. ODA deficiency was diagnosed when < 3 ODAs were visible, partial ODA deficiency when <7 ODAs were observed. IDA deficiency was defined as <1.5 IDA per cilium. Absence or displacement of the central pair was indicative of PCD if present in >1/3 of the cilia. SCD score was calculated as a composite score of membrane abnormalities (blebs, naked cilia, compound cilia), disorganization of microtubules, abnormalities of the central microtubular pair (absent, transposed, eccentric, double) and peripheral microtubular abnormalities (absent, supplementary, dislocated) [29]. SCD score >5% was defined as abnormal.

Every biopsy sample was cultured irrespective of biopsy results [30]. Cells were first grown in a monolayer to expand the basal cell population without cilia (dedifferentiation). Cells were then transferred to a suspension medium to induce redifferentiation into ciliated epithelial cells after 2–3 weeks (ciliogenesis). Ciliary activity (coordination and CBF) and TEM were reevaluated after cell culture. Abnormalities secondary to respiratory infection and toxic substances thus disappeared and SCD and PCD could be clearly distinguished [29]. Coordination of the ciliary activity was subjectively evaluated. Normal coordination implied the observation of rotation of spheroids and movement of debris in the medium due to the beating motion of the cilia. PCD was defined as the absence of coordinated ciliary activity after ciliogenesis in culture, indicating that efficient ciliary transport was absent (see Additional files 1, 2 and 3 for examples of normal and abnormal coordination of ciliary activity). The validity of this evaluation of coordination to distinguish PCD from SCD was replicated by an independent group [31].

If patients consented and if DNA was available, genetic analysis was performed in a subgroup of patients with NU. Exome sequencing was performed to detect mutations in *DNAH11* and 29 other candidate genes (Additional file 4) (DNA library preparation kit TruSeq DNA Sample Prep kit v2, Illumina; Exome capturing kit SeqCap EZ Human Exome Library v2.0, Nimblegen; Sequencing kits TruSeq PE Cluster Kit v3-cBot-HS and TruSeq SBS Kit v3-HS, Illumina; Sequencing apparatus Hiseq 2000, Illumina; Bioinformatic analysis CLC Genomics Workbench, CLC Bio). It should be noted that only a ‘partial’ exome was obtained and that any mutation located in a gene not covered by the partial exome, could therefore not be identified. Sanger sequencing, performed by amplifying and sequencing the coding exons and flanking intronic sequences of *DNAH11*, was performed in most patients to confirm the results of exome sequencing.

### Statistical analysis

Clinical characteristics were compared between PCD with NU and PCD with dynein deficiencies (DD) (partial or complete ODA deficiency or a combination of IDA and ODA deficiency) and PCD with central pair abnormalities (CP).

A stratified analysis was performed per age group (children below age 18 and adults from age 18 years on) and according to presence of situs inversus and infection with Pseudomonas.

Mann–Whitney U or Kruskal-Wallis test were used for continuous variables when data were not normally distributed. If there was a significant difference, paired subgroup analysis was performed to identify which of the groups was different. Proportions between groups were compared using chi square statistics. Biometry and lung function data were compared to the normal reference values using one-sample t-test.

Functional analysis before and after cell culture was compared using related samples Wilcoxon signed rank test.

A p-value <0.05 was defined as statistically significant. SPSS 21.0 (IBM Corp., Armonk, NY) was used for statistical analyses.

## Results

Between January 1990 and August 2012, PCD was diagnosed in 206 patients. Ninety patients were diagnosed with PCD with DD (44%)(45 ODA deficiency, 37 partial ODA deficiency and 8 IDA and ODA deficiency). PCD with NU was diagnosed in 68 patients (33%), and PCD with CP in 41 patients (20%) (22 with eccentric central pair and IDA deficiency, 19 with absence of central pair). PCD due to ciliary aplasia (n = 6) and peripheral microtubular abnormalities (n = 1) was rare. Therefore, these patients were excluded for further analysis. Of the remaining 199 patients, no clinical data could be obtained in 31. Data from 168 remaining patients were included for analysis.

### Clinical characteristics in patients with PCD and comparison between NU and other PCD types

#### Lifetime prevalence of symptoms

Clinical characteristics of the total PCD group and of the 3 subpopulations are reported in Table 1. Lifetime prevalence of most symptoms did not differ between the subgroups. SI was significantly less frequent in the NU and CP group compared to the DD group (p <0.05). North African ancestry was more common in the CP subgroup, and this was linked to higher incidence of consanguinity (p <0.05).

**Table 1:** Lifetime prevalence of patient characteristics during follow-up

		**Total population (n = 168)**	**Normal ultrastructure (n = 59)**	**Dynein deficiency (n = 74)**	**Central pair abnormalities (n = 35)**	**p-value for comparison between groups**
General	Age at diagnosis in years (median - IQR)	9.9 (3.7–23.4)	10.2 (4.4–21.8)	10.8 (3.7–29.6)	8.7 (2.2–19.3)	0.60
	Male gender, n (%)	91 (54.8)	32 (54.2)	39 (53.4)	20 (58.8)	0.85
	North African ancestry, n (%)	38 (23.3)	8 (14.0)	12 (16.7)	18 (52.9)*	**0.0001**
	Consanguinity, n (%)	33 (19.6)	9 (15.3)	12 (16.2)	12 (34.3)*	**0.004**
	Sibling with PCD, n (%)	37 (22.0)	9 (15.3)*	17 (23.0)	11 (31.4)*	**0.047**
	Situs inversus, n (%)	69 (41.1)	18 (30.5)	42 (56.8)*	9 (25.7)	**0.001**
	Structural cardiac abnormality, n(%)	11 (6.5)	4 (8.7)	5 (9.6)	2 (9.1)	0.75
Lower respiratory tract	Neonatal respiratory problems, n (%)	75 (44.6)	27 (45.8)	30 (40.5)	18 (51.4)	0.60
	Wheezing, n (%)	79 (47.0)	28 (46.5)	37 (50.0)	14 (40.0)	0.33
	Bronchiectasis, n (%)	114 (67.9)	40 (67.8)	51 (68.9)	23 (65.7)	0.51
	Lobar collapse, n (%)	68 (40.5)	23 (39.0)	30 (40.5)	15 (42.9)	0.16
	Pulmonary infiltrate, n (%)	100 (59.5)	37 (62.7)	43 (58.1)	20 (57.1)	0.29
	Lobectomy, n (%)	15 (8.9)	4 (6.8)	7(9.5)	4 (11.4)	0.49
	*H. influenzae*, n (%)	72 (42.9)	28 (47.5)	33 (44.6)	11 (31.4)	0.15
	*Strep. pneumoniae*, n (%)	45 (26.8)	18 (30.5)	20 (27.0)	7 (20.0)	0.08
	*Staph. aureus*, n (%)	28 (16.7)	9 (15.3)	17 (23.0)	2 (5.7)	0.07
	*Pseud. aeruginosa*, n (%)	27 (16.1)	9 (15.3)	14 (18.9)	4 (11.4)	0.59
Upper respiratory tract	Recurrent sinusitis, n (%)	111 (66.1)	41 (69.5)	52 (70.3)	18 (51.4)	0.67
	Nasal polyps, n (%)	54 (32.1)	19 (32.2)	25 (33.8)	10 (28.6)	0.80
	Sinus surgery, n (%)	68 (40.5)	27 (45.8)	31 (41.9)	10 (28.6)	0.79
	nNO (ppb) (median – IQR)	52 (30–170)	64 (37–199)	40 (25–98)	89 (24–327)	0.15
	Ear discharge, n (%)	70 (41.7)	30 (50.8)	26 (35.1)	14 (40.0)	0.33
	Ear drum perforation, n (%)	35 (20.8)	15 (25.4)	11 (14.9)	9 (25.7)	0.15
	Hearing loss, n (%)	58 (34.5)	20 (33.9)	30 (40.5)	8 (22.9)	0.33
	Hearing aid, n (%)	10 (6.0)	2 (3.4)	5 (6.8)	3 (8.6)	0.38
	Grommets insertion, n (%)	82 (48.8)	30 (50.8)	33 (44.6)	19 (54.3)	0.56
	Adenotomy, n (%)	65 (38.7)	28 (47.5)	26 (35.1)	11 (31.4)	0.76

Legend: Table 1 shows general patient characteristics, incidence of specific lower and upper respiratory tract signs and symptoms in the total PCD group and in the 3 subgroups. P-values for the comparison between the three subgroups (Kruskal-Wallis for continuous variables and χ^2^ for proportions) are shown in the last column. If they revealed a significant result, pairwise comparisons were performed and the significant outlier was identified and marked with *.

*IQR*, inter quartile range.

*nNO*, nasal nitric oxide.

*PCD*, primary ciliary dyskinesia.

Data marked in bold denote significant differences.

Median age at diagnosis in the NU group was 10.2 years (IQR 4.4-21.8, range 0.0-61.9), two third of the patients being diagnosed during childhood. Median age at diagnosis in pediatric patients was 5.1 years (IQR 2.0-9.6, range 0.0-14.9). There was no significant difference in age at diagnosis between patients with NU, with or without SI (p 0.71) (10.0 years, IQR 3.0-20.2, range 0.0-61.9 and 12.7 years, IQR 5.1-23.5, range 0.0-58.2, respectively).

A history of neonatal respiratory problems was reported in 28 (47.5%) patients with NU. It was higher in children with NU (n = 20/32, 63%) than in adults with NU (n = 8/27, 29.6%) (p 0.012). Bronchiectasis was more frequent in adults with NU (n = 24/27, 89%) than in children with NU (n = 16/32, 50%) (p 0.001). Nasal polyps were more prevalent in adults (n = 14/27, 51.9%) compared to children (n = 5/32, 115.6%) (p 0.003) with NU. Nevertheless, 8/29 (27.6%) children had already undergone sinus surgery for nasal polyps or recurrent sinusitis.

Nasal nitric oxide levels were available for 88/168 patients (41 with DD, 34 with NU and 13 with CC). There was no difference between the subgroups. Normal values at 300 ppb cut-off were observed in 7/34 patients with NU, 6/41 with DD and 4/13 with CC (p 0.231).

#### Clinical characteristics at last follow-up

Clinical characteristics at last follow-up are shown in Table 2. These did not differ between patients with NU and those with DD or CP.

**Table 2:** Patient characteristics at last follow-up

	**Total (n = 168)**	**Normal ultrastructure (n = 59)**	**Dynein deficiency (n = 74)**	**Central pair abnormalities (n = 35)**	**p-value for comparison between groups**
Age in years (median - IQR)	17.7 (9.5–28.1)	15.8 (9.7–26.7)	19.6 (10.9–31.9)	14.2 (6.7 – 24.5)	0.14
Years since diagnosis (median – IQR)	4.3 (0.0–11.4)	4.3 (0.4–9.0)	3.6 (0.0–12.8)	5.3 (0.0–11.5)	0.88
Weight z-score (mean - SD)	−0.12 (1.25)	−0.25 (1.25)	0.07 (1.33)	−0.51 (0.97)*	0.16
Height z-score (mean - SD)	−0.63 (1.23)*	−0.53 (1.14)*	−0.64 (1.26)*	−0.91 (1.32)*	0.67
BMI z-score (mean - SD)	0.15 (1.23)	0.02 (1.17)	0.41 (1.22)	−0.25 (1.31)	0.10
Chronic cough, n (%)	136 (81.0)	48 (81.4)	65 (87.8)	23 (65.7)	0.13
Chronic sputum production, n (%)	113 (81.9)	43 (81.1)	52 (83.9)	18 (78.3)	0.83
Clubbing, n (%)	31 (18.5)	10 (16.9)	16 (21.6)	5 (14.3)	0.57
Nasal secretions, n (%)	136 (81)	49 (83.1)	60 (81.1)	27 (77.1)	0.63
FEV_1_ z-score (median - IQR) (n = 112)	−1.79 (−2.96; –0.76)*	−1.47 (−2.67; –0.47)*	−1.94 (−3.13; –1.19)*	−2.34 (−3.61; –0.55)*	0.17
FVC z-score (median - IQR) (n = 112)	−0.76 (−1.61; –0.15)*	−0.54 (−1.43; 0.06)*	−0.98 (−1.77; –0.21)*	−0.77 (−2.05; –0.30)*	0.75

Legend: Table 2 shows clinical status at last follow-up in the total patient group and in the 3 subgroups. P-values for the comparison between the three subgroups (Kruskal-Wallis for continuous variables and χ^2^ for proportions) are shown in the last column. * marks results that are significantly different from the reference population. For FEV_1_ and FVC the total number of patients was only 112 because some patients were too young (<6 years) to perform spirometry (n = 25) or because spirometry results were not available.

*BMI*, body mass index.

*FEV*_*1*_, forced expiratory volume in one second.

*FVC*, forced vital capacity.

*IQR*, inter quartile range.

*SD*, standard deviation.

Current median age of the total group was 17.7 years and this was similar in all subgroups. Weight and BMI of the total group did not differ from that of the Flemish reference population. Height was significantly lower compared to that of healthy Flemish subjects (mean z-score −0.53, 95% CI −0.85;-0.22, p 0.001). This was also the case in the subgroups. Even when patients with North African origin were excluded from analysis, the mean z-score and 95% CI for height were still lower than zero (i.e. -0.49, 95% CI −0.84; -0.15, p 0.006).

Mean FEV_1_ and FVC in patients with NU were lower than normal (mean FEV_1_ z-score −1.45, 95% CI −1.90; -1.01; mean FVC z-score −0.82, 95% CI −1.22; -0.42), even when only children (n = 21) were evaluated (mean FEV_1_ z-score −1.6, 95% CI −2.11; -1.1; mean FVC z-score −0.86, 95% CI −1.28). The same was true in the other subgroups. In 17/45 (38%) of the patients with NU, FEV_1_ z-score was below −2, in 21/45 (47%) between −2 and 0 and in 15% between 0 and 2. In this cross-sectional evaluation, FEV_1_ z-score did not worsen with age (−0.02 z-score per year for NU, 95% CI −0.049; 0.017, p 0.325), but was already abnormal at the age of 5. For a graphical illustration of the lung function data, see Additional file 5.

In the total group of PCD patients, the *Pseudomonas* positive patients were significantly older (n = 28, median age 23.6 y) (p 0.017) and had significantly lower FEV_1_ z-scores (median FEV_1_ z-score −2.2) (p 0.032) than the *Pseudomonas* negative patients (n = 140, median age 19.1 years, median FEV_1_ z-score −1.5). Chronic *P. aeruginosa* infection was seen in 3 patients with NU, 3 patients with DD and 2 patients with CP, all but one adults.

### Ciliogenesis in culture and the diagnosis of PCD

Two hundred and six patients were diagnosed with PCD and thus had abnormal ciliary coordination after cell culture. This amounted to 6.5% of the 3190 evaluated samples. In 75% of biopsies performed, the technique of ciliogenesis in culture was successful at first biopsy.

Results for functional and structural evaluation before and after ciliogenesis in culture for the total group and the 3 subgroups are presented in Table 3. In 81/199 (40.7%) patients, no cilia were found for functional evaluation on biopsy, either because of insufficient material available or because cilia were not detected due to extreme SCD.

**Table 3:** Results of functional and ultrastructural evalution before and after cell culture

		**Normal values**	**Normal ultrastructure (n = 68)**		**Dynein deficiency (n = 90)**		**Central pair abnormalities (n = 41)**		**p-value for comparison between 3 groups**	
			**Before**	**After**	**Before**	**After**	**Before**	**After**	**Before**	**After**
SAMPLE QUALITIY	No cilia found									
	- For functional evaluation		30 (44%)	0	36 (40%)	0	15 (37%)	0	0.782	NA
	- for TEM		22 (32%)	0	27 (30%)	0	13 (32%)	0	0.948	NA
	Number of cilia counted for TEM		82 (45–114)	85 (59–132) *0.236*	92 (58–160)	84 (49–129) *0.839*	70 (52–126)	95 (61–125) *0.194*	0.490	0.620
	Total SCD (%)	<5%	10 (5–22)	1 (0–4) *0.0001*	13 (7–21)	1 (0–4) *0.0001*	52 (31–70)*	52 (27–68)* *0.612*	0.0001	0.0001
SPECIFIC MEASUREMENTS	CBF (Hz) (median-IQR)	7.9 (1.8) (mean, SD)	5.8 (0–9.6)	0 (0–6.3) *0.001*	0 (0–0)*	0 (0–0)* *0.674*	5 (0–6.9)	4.4 (0–5.6) *0.170*	0.0001	0.0001
	N(%) of patients with normal CBF		18 (27%)	19 (28%)	4 (4%)*	11 (12%)*	14 (34%)	21 (51%)	0.0001	0.0001
	N(%) of patients with Normal coordination		17 (25%)	0	2 (2%)*	0	8 (20%)	0	0.0001	NA
	N(%) of patients with normal CBF and coordination		11 (16.2%)	0	1 (1.1%)*	0	6 (14.6%)	0	0.002	NA
	N of ODA (median-IQR)	8.4 (0.8) (mean, SD)	8.3 (7.8–8.6)	8.4 (7.9–8.7) *0.238*	2.3 (1.35–4.1)*	2.4 (1.4–4.6)* *0.233*	8.6 (8.3–8.9)	8.6 (8.3–8.7) *0.221*	0.0001	0.0001
	N of IDA (median-IQR)	3.7 (1.4) (mean, SD)	3.2 (2.7–3.7)*	2.7 (2.3–3)* *0.001*	2.9 (2.4–3.5)	2.1 (1.7–2.6) *0.0001*	3.0 (1.3–3.6)	2.3 (1.1–2.7) *0.0001*	0.036	0.0001

Legend: Sample specifications and results of functional (CBF and coordination of ciliary activity) and structural evaluation of the cilia before and after cell culture in the 3 subgroups. Normal values are reported in the first column, based on our own laboratory references [18,32], and updated with more recent data. Each subsequent column represents a subgroup of PCD and each column is subdivided in a first column for evaluation before cell culture (= in the biopsy) and a second column after cell culture. Individual results were compared before and after ciliogenesis (Wilcoxon signed rank test), and the result of these comparisons is marked in italic. Comparison between the 3 PCD subgroups (Kruskal-Wallis or χ^2^ test) is presented in the last table. If the comparison revealed a significant result, pairwise comparisons were performed and the significant outlier was identified and marked with *.

*CBF*, ciliary beat frequency.

*IDA*, inner dynein arm.

*IQR*, inter quartile range.

*NA*, not appropriate.

*NU*, normal ultrastructure.

*ODA*, outer dynein arm.

*PCD*, primary ciliary dyskinesia.

*SCD*, secondary ciliary dyskinesia.

*SD*, standard deviation.

*TEM*, transmission electron microscopy.

In the NU subgroup, functional ciliary analysis was possible before as well as after ciliogenesis in 38 of 68 (56%) patients. In 11/38 evaluation of both ciliary coordination and CBF were assessed as normal in the biopsy (16% of patients with final diagnosis of NU). However, they were abnormal after cell culture. If only data prior to cell culture had been used, diagnosis of PCD with NU would have been missed in at least 11 patients, representing 5% of total PCD diagnoses. Repeat biopsy was performed in 6 of these 11 patients (3 patients had 2 biopsies, 2 patients had 3 biopsies and 1 patient had 5 biopsies). All repeat biopsies confirmed absence of ciliary coordination after ciliogenesis and thereby confirmed the diagnosis of PCD with NU. nNO was normal in only 1 of these patients, low in 5 and missing in another 5 of these 11 patients.

The same discrepancy between functional evaluation before and after cell culture was observed in PCD with CP, but rarely in PCD with DD (see Table 3). SCD disappears after culture in the NU and DD group, but not in the CP group. This is because the absence or displacement of the central microtubular pair is in itself a marker of SCD. IDA counts in the NU group did not significantly differ from a non-PCD population (p 0.374).

In the NU group, CBF after culture was significantly lower than in the biopsy (p 0.001), indicating that ‘hyperfrequent’ beating disappears after ciliogenesis. This discrepancy was not observed in the CP (p 0.674) and DD group (p 0.170).

Genetic analysis of *DNAH11* was performed in 29 of 68 patients with PCD and NU, including 6/11 patients with discordant findings pre versus post ciliogenesis. In total, PCD with NU could be confirmed genetically in 23/29 patients (79%) or in 19/25 families (76%). Biallelic *DNAH11* mutations were found in 21 patients, biallelic *HYDIN* mutations in one patient and a homozygous *CCDC103* mutation in one other patient. The latter mutation was previously described as a hypomorphic mutation causing no abnormalities on TEM, but functional abnormalities concordant with a diagnosis of PCD [6]. In two patients, only one *DNAH11* mutation could be found. No mutations could be found in only 4/29 (14%) patients. See Additional file 6 for detailed information on the results of exome sequencing.

## Discussion

We described the clinical characteristics of our total population of patients with PCD, and focused on the subgroup of patients with PCD and NU. In addition, we described the diagnostic pathway needed in order not to miss PCD with NU. Of the 206 Belgian patients with PCD, patients with NU emerged as an important subgroup.

In patients with PCD and NU, clinical characteristics were similar compared to those of patients with PCD and DD or CP. Our analysis confirmed that PCD with NU have severe clinical symptoms: low height for age, chronic cough and sputum production, abnormal lung function from an early age on and high prevalence of bronchiectasis. The prevalence of bronchiectasis and nasal polyps is higher in adults than in children, meaning that the disease is progressive over time.

In our population of PCD with NU, the incidence of SI was significantly lower than 50%, as was the case in PCD with CP. The group of PCD with CP was a combination of patients with absence of the central pair (*RSPH4A* or *RSPH9* mutations) in whom SI did not occur and patients with displacement of the central pair (*CCDC39* or *CCDC40* mutations) in whom SI was found. Similarly, the group of PCD with NU could be a combination of patients with NU and patients with subtle CP. Indeed, subtle abnormalities in the central pair that cannot be visualized by TEM were recently reported by Olbrich et al. [11], who described *HYDIN* mutations as a cause of PCD with NU without SI. One patient in our population with NU had 2 *HYDIN* mutations.

Recent data of the European Taskforce on PCD demonstrated a median age at diagnosis of 5.3 years for patients with a current age below 20 years [33]. When only patients younger than 18 years were included, the age at diagnosis in our center was similar (5.1 years). However, one third of our population (59/168) was only diagnosed in adulthood. This could be an indication that the diagnosis can easily be missed or that patients with a milder form only present towards adulthood.

Our cross-sectional data did not show any difference in FEV_1_ or FVC between ages. Remarkably, however, lung function was already found to be abnormal at the age of 5 years. This observation suggests that destruction of the lung parenchyma starts early, as previously suggested [34,35]. In addition, since complications like bronchiectasis are more frequent in older patients, disease progression is most likely. Further prospective research in a larger patient population is necessary to corroborate these findings.

To demonstrate the need for cell culture to robustly diagnose PCD with NU, we retrospectively analyzed our diagnostic algorithm. We have clearly shown that PCD with NU (and also with CP) can be missed if cell culture is not an integral part of the diagnostic path. To date, a combination of functional evaluation of the ciliary motility and TEM in a fresh biopsy sample has been the gold standard to detect PCD with or without ultrastructural abnormalities. Cell culture was to be reserved to diagnose PCD in selected, difficult cases [36,37]. We have now shown that ciliogenesis in culture is essential not to miss the subgroup of PCD with NU: in the current population, 16% of the patients with PCD and NU would have been missed (5% of the total PCD population). In a subset of patients, the features of ciliary dysmotility only became apparent after ciliogenesis in culture. Thus, cell culture increases both diagnostic specificity and sensitivity. Moreover, we have identified several new mutations in *DNAH11* in patients with NU. This confirms that the culture technique is capable of correctly detecting PCD with NU. Although no functional testing was performed, most mutations were likely to be pathogenic, since most of them were either stop codon or frameshift mutations.

We agree that it is difficult to understand that coordination is normal in a fresh biopsy sample and reverts to abnormal after culture. We hypothesize that some growth factors might be absent in the suspension medium, which alters a hyperfrequent beating phenotype turn into a ciliostatic phenotype, leading to uncoordinated ciliary motion. We are confident that cell death did not occur, because cilia only reappeared on the last few days of suspension culture. Misinterpretation is unlikely, as the result could be confirmed repeatedly. Pifferi et al. have recently shown the same phenomenon of normal evaluation of ciliary coordination in the fresh biopsy and abnormal ciliary coordination after culture in one patient with 2 *DNAH11* mutations [31]. Also for patients with CP, we found a high incidence of normal functional evaluation in the fresh biopsy. Because central pair abnormalities are also a sign of SCD, cell culture has an added value in PCD with CP. Therefore, a repeat biopsy with a cell culture procedure must be considered when clinical suspicion of PCD is high and an alternative diagnosis is lacking.

One of the strengths of our study is population size. A group of patients with PCD and NU has also been described by Knowles et al. [16], but this study focused on genetic diagnosis.

This study has some weaknesses. Although we did measure CBF, we did not perform a computerized three-dimensional ciliary beat pattern analysis, proposed as gold standard by some centers [22]. It is impossible to perform this technique on moving spheroids. Therefore, we were not able to link the ultrastructural abnormalities to a specific beat pattern. However, ciliary beat pattern analysis is difficult to interpret and abnormalities may be very subtle [38]. Variability of beat pattern analysis before versus after culture is another concern [38]. We did not systematically include nNO measurement as a diagnostic technique, because it was not available in most of the referring centers. To date however, nNO measurements have not been standardized and need further validation.

We did not detect PCD with isolated IDA deficiency. IDA deficiency is difficult to detect [39] and its existence is debated [40]. Moreover, no gene mutations have been described causing isolated IDA deficiency. It can be hypothesized that some of the IDA deficiencies might be misinterpreted as NU, but there was no significant difference in IDA counts between PCD with NU and control patients without PCD. The fact that we did not find isolated IDA deficiency after cell culture, might confirm that it is part of SCD, as suggested previously [40].

One could question the usefulness of TEM in the diagnosis of PCD, as it does not add much information on disease phenotype. TEM is expensive, time-consuming and difficult to interpret. Additionally, it is insufficient as stand-alone test since some abnormalities can be very subtle. However, if genetic testing becomes routine in the future, TEM morphology may guide genetic investigations. Genetic analysis will aid diagnosis and will allow to examine genotype-phenotype correlations.

## Conclusion

We have analyzed the clinical characteristics of a large population with PCD. PCD with NU emerges as a large subgroup and does not differ considerably from other subgroups. We have shown the need for ciliogenesis in culture so as not to miss PCD with NU. The robustness of these diagnoses of PCD with NU is further proven by the consistency of the findings in repeat biopsies as well as by demonstration of mutations by genetic analysis.

## Abbreviations

CF: Cystic fibrosis; CP: Central pair abnormalities; DD: Dynein deficiency; DNAH: Dynein axonemal heavy chain; ENT: Ear nose, throat; FEV1: Forced expiratory volume in one second; FVC: Forced vital capacity; IDA: Inner dynein arm; nNO: Nasal nitric oxide; NU: Normal ultrastructure; ODA: Outer dynein arm; PCD: Primary ciliary dyskinesia; SCD: Secondary ciliary dyskinesia; SI: Situs inversus; TEM: Transmission electron microscopy.

## Competing interests

The authors declare that they have no competing interests.

## Authors’ contributions

MB, AS and KDB contributed to the design of the study, the collection and analysis of the data and writing of the manuscript. HC and MJA contributed to analysis of the genetic results, and reviewed the manuscript. MP, LJD, FLV, SVD, AM, VG and MJO contributed to collection of the data and reviewed the manuscript. All authors gave final approval for the manuscript.

## Supplementary Material

Additional file 1 **Example of coordinated ciliary activity.** Coordinated ciliary movement can be observed: there is rotation of spheroids in one direction. Click here for file

Additional file 2 **Example of coordinated ciliary activity.** Coordinated ciliary movement can be observed: there is active movement along the ciliary border of a red blood cell. Click here for file

Additional file 3 **Example of uncoordinated ciliary activity.** Uncoordinated ciliary movement can be observed: there is no movement of the spheroids, nor movement of debris or red blood cells in the medium. Some cilia are static, others beat with a ‘hyperfrequent’ pattern. Click here for file

Additional file 4 List of candidate genes that were used for exome sequening analysis. Click here for file

Additional file 5 **Cross sectional data of FEV**_**1**_** (a) and FVC (b) in the subgroups according to age.** Lung function results were available from the age of 5 years. Results were available for 45 patients with NU, 51 patients with DD and 16 patients with CP. Different symbols refer to different subtypes. Shaded areas denote −2 to +2 z-scores. The full lines represent the regression line for the total group. Click here for file

Additional file 6 **Results of the exome sequencing analysis in 25 families with PCD and NU.** Most of the mutations found, had not previously been described in the literature (previously reported mutations are underlined). Most of the mutations are likely to be pathogenic because they are frameshift and/or stopcodon mutations (marked with *). The other mutations were not previously reported as SNP (SNP137–Common Variations database) and were thus also likely pathogenic. It should be noted that we cannot prove the pathogenicity of some of these mutations. In the family of DWS and DB, the asymptomatic father was found to be heterozygous for the p.Arg2900* mutation, the asymptomatic mother heterozygous for the c.10568 + 1G > A mutation, and an asymptomatic sister was heterozygous for the c.10568 + 1G > A mutation. This mutation involves the most important nucleotide of a splice site and therefore is very likely a splice-site mutation. The sister of VDE without PCD (confirmed on nasal biopsy) only carried the p.Arg1834Gly mutation. The p.Arg2068His and p.Trp3238Arg mutations have been found in 3 patients with PCD. The p.Arg2068His and p.Trp3238Arg mutations are therefore very likely two mutations in cis. Only one of these two mutations might be pathogenic. The updated coding region and mutation nomenclature was used [16]. ° only Sanger sequencing was performed, no exome sequencing. Marked in grey: Patients with PCD with normal ultrastructure and normal evaluation in biopsy. SS: situs solitus. SI: situs inversus. ND: not done. Click here for file
