# Supplementary material for: Primary ciliary dyskinesia: critical evaluation of clinical symptoms and diagnosis in patients with normal and abnormal ultrastructure
Source: Orphanet J Rare Dis. 2014 Jan 22;9:11. doi: 10.1186/1750-1172-9-11 (PMC4016480; doi:10.1186/1750-1172-9-11)
Supplement: Additional file 4 — List of candidate genes that were used for exome sequening analysis. [file 1750-1172-9-11-S4.docx]

| DNAH11 | dynein, axonemal, heavy chain 11 |
| --- | --- |
| DNAH5 | dynein, axonemal, heavy chain 5 |
| DNAI1 | dynein, axonemal, intermediate chain 1 |
| DNAI2 | dynein, axonemal, intermediate chain 2 |
| C14orf104 | chromosome 14 open reading frame 104 (=KTU) |
| LRRC50 | leucine rich repeat containing 50 |
| RSPH9 | radial spoke head 9 homolog (Chlamydomonas) |
| RSPH4A | radial spoke head 4 homolog A (Chlamydomonas) |
| TXNDC3 | thioredoxin domain containing 3 (spermatozoa) |
| WDR63 | WD repeat domain 63 |
| CCDC39 | coiled-coil domain-containing protein 39 |
| CCDC40 | coiled-coil domain-containing protein 40 |
| RPGR | retinitis pigmentosa GTPase regulator |
| OFD1 | oral-facial-digital syndrome 1 |
| WDR69 | WD repeat domain 69 |
| IFT88 | intraflagellar transport 88 homolog (Chlamydomonas) |
| DNAH7 | dynein, axonemal, heavy chain 7 |
| SPEF2 | SPEF2 sperm flagellar 2 |
| DNAL1 | dynein, axonemal, light chain 1 |
| AK7 | adenylate kinase 7 |
| DNAAF3 | dynein, axonemal, assembly factor 3 (DNAAF3 = PF22) |
| CCDC103 | coiled-coil domain-containing protein 103 |
| HYDIN | HYDIN, axonemal central pair apparatus protein |
| HEATR2 | Heat repeat-containing protein 2 |
| LRRC6 | Leucin-rich repeat-containing protein 6 |
| CCDC114 | Coiled-coil domain-containing protein 114 |
| CCDC65 or DRC2 | Coiled-coil domain-containing protein 65 |
| CCDC164 or DRC1 | Coiled-coil domain-containing protein 164 |
| ARMC4 | Armadillo repeat motifs containing 4 |
| DYX1C1 | Dyslexia susceptibility 1 candidate 1 |
