# Supplementary figures and images for: Primary ciliary dyskinesia: critical evaluation of clinical symptoms and diagnosis in patients with normal and abnormal ultrastructure
Source: Orphanet J Rare Dis. 2014 Jan 22;9:11. doi: 10.1186/1750-1172-9-11 (PMC4016480; doi:10.1186/1750-1172-9-11)

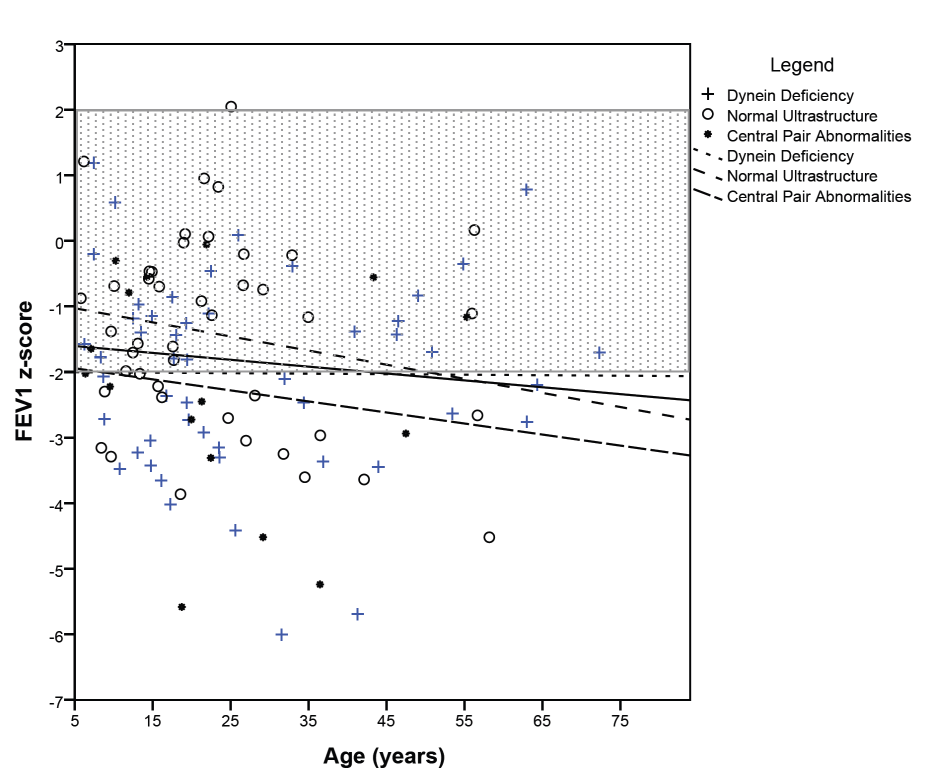
a)


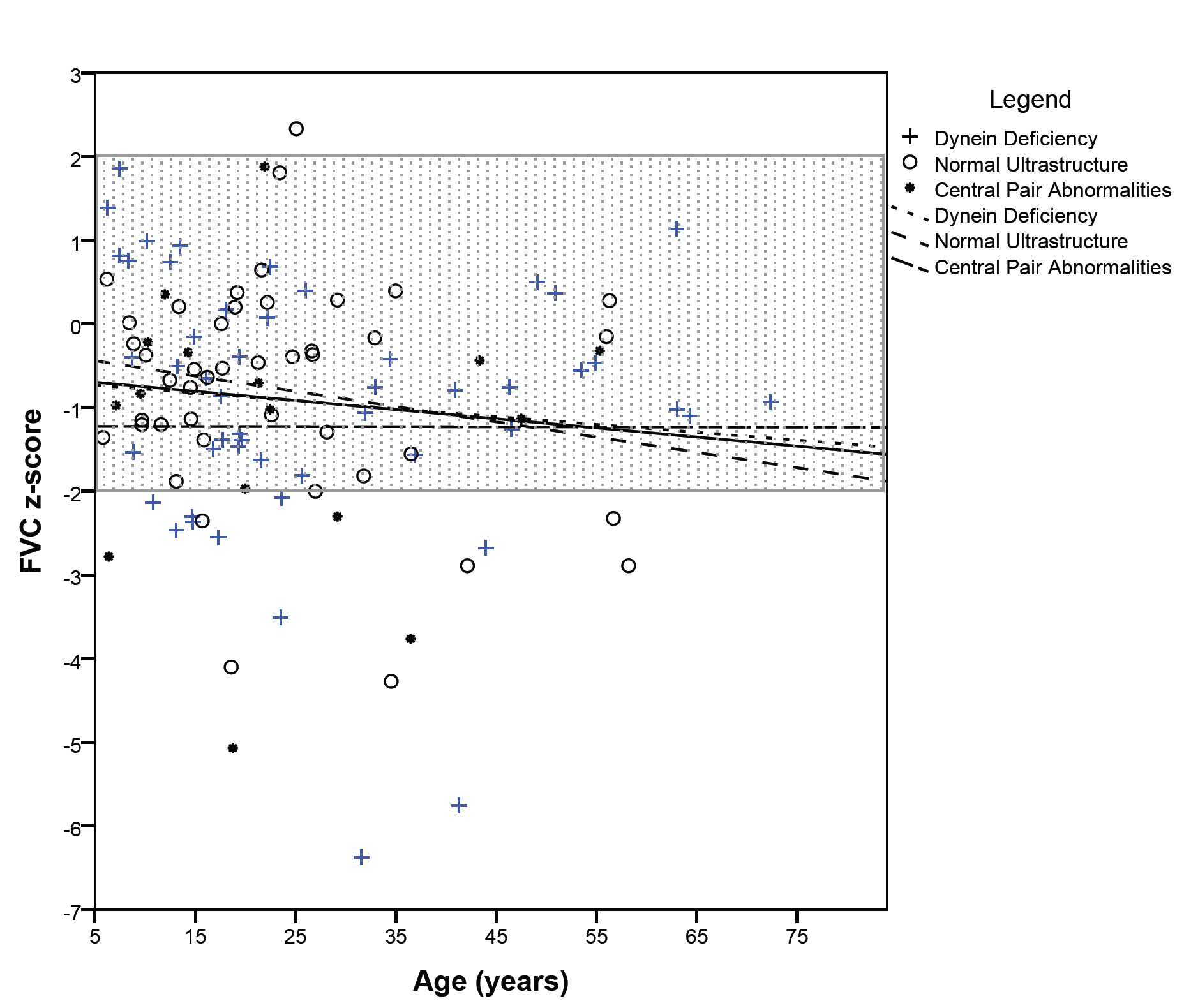


b)

Supplement: Additional file 5 — Cross sectional data of FEV1 (a) and FVC (b) in the subgroups according to age. Lung function results were available from the age of 5 years. Results were available for 45 patients with NU, 51 patients with DD and 16 patients with CP. Different symbols refer to different subtypes. Shaded areas denote −2 to +2 z-scores. The full lines represent the regression line for the total group. [file 1750-1172-9-11-S5.docx]
