# Supplementary material for: Primary ciliary dyskinesia: critical evaluation of clinical symptoms and diagnosis in patients with normal and abnormal ultrastructure
Source: Orphanet J Rare Dis. 2014 Jan 22;9:11. doi: 10.1186/1750-1172-9-11 (PMC4016480; doi:10.1186/1750-1172-9-11)
Supplement: Additional file 6 — Results of the exome sequencing analysis in 25 families with PCD and NU. Most of the mutations found, had not previously been described in the literature (previously reported mutations are underlined). Most of the mutations are likely to be pathogenic because they are frameshift and/or stopcodon mutations (marked with *). The other mutations were not previously reported as SNP (SNP137–Common Variations database) and were thus also likely pathogenic. It should be noted that we cannot prove the pathogenicity of some of these mutations. In the family of DWS and DB, the asymptomatic father was found to be heterozygous for the p.Arg2900* mutation, the asymptomatic mother heterozygous for the c.10568 + 1G > A mutation, and an asymptomatic sister was heterozygous for the c.10568 + 1G > A mutation. This mutation involves the most important nucleotide of a splice site and therefore is very likely a splice-site mutation. The sister of VDE without PCD (confirmed on nasal biopsy) only carried the p.Arg1834Gly mutation. The p.Arg2068His and p.Trp3238Arg mutations have been found in 3 patients with PCD. The p.Arg2068His and p.Trp3238Arg mutations are therefore very likely two mutations in cis. Only one of these two mutations might be pathogenic. The updated coding region and mutation nomenclature was used [16]. ° only Sanger sequencing was performed, no exome sequencing. Marked in grey: Patients with PCD with normal ultrastructure and normal evaluation in biopsy. SS: situs solitus. SI: situs inversus. ND: not done. [file 1750-1172-9-11-S6.docx]

| Patient(s) | Situs | Gene | Allele 1 | Allele 2 | Third mutation | Confirmation by Sanger |
| --- | --- | --- | --- | --- | --- | --- |
| HCMJ92 | SS | DNAH11 | p.Lys896fs | p.Lys896fs |  | yes |
| KJ | SI | DNAH11 | p.Arg2900* | p.Glu3146* |  | yes |
| HCMJ95 | SS | DNAH11 | p.Pro959fs | p.Glu3146* |  | yes |
| DWS and DB° | SI/SS | DNAH11 | p.Arg2900* | c.10568+1G>A |  | yes |
| VDE | SS | DNAH11 | p.Arg2900* | p.Arg1834Gly |  | yes |
| HCMJ101 | SS | DNAH11 | p.Arg1445* | p.Arg2068His | p.Trp3238Arg | yes |
| HCMJ110 | SI | DNAH11 | p.Phe2641Ser | p.Arg2068His | p.Trp3238Arg | yes |
| BC4 and HCMJ91 | SS/SI | DNAH11 | c.11968-1G>C | c.11968-1G>C |  | yes |
| CI | SS | DNAH11 | c.7812-2A>T | p.Val2518Ile (=common SNP135, rs68023059, avHet=0.073965) |  | yes |
| SE and SA | SS/SS | DNAH11 | c.9103-2A>C | p.Arg4437Cys |  | yes |
| HCMJ105 | SI | DNAH11 | p.Arg989* | p.Leu4120Phe |  | yes |
| HCMJ108 | SI | CCDC103 | p.His154Pro | p.His154Pro |  | ND |
| VEV | SS | HYDIN | p.Glu41* | p.Arg1059* |  | ND |
| HCMJ137 and VR° | SS/SS | DNAH11 | p.Arg2997Gln | p.Arg3580His |  | yes |
| HCMJ2 | SS | DNAH11 | p.Trp904* | p.Arg1445* |  | yes |
| HCMJ134 | SI | DNAH11 | Arg1834Gly | 0 |  | yes |
| HCMJ20° | SI | DNAH11 | c.[122_147del26 | c.[11853delC] |  | yes |
| HCMJ7° | SS | DNAH11 | c.[13472_13541dup70] (duplication -> frameshift) | p.Arg2068His | p.Trp3238Arg | yes |
| HCMJ143 | SS | DNAH11 | p.Cys2756Arg | p.His2788Asp |  | yes |
| HCMJ133 | SI | DNAH11 | p.Ser1119Asn | p.Phe4266_Asn4267delinsIle | p.Trp1222Arg | ND |
| DiSh | SS | DNAH11 | Pro4183Leu | 0 |  | ND |
| CS | SS |  | 0 | 0 |  | 0 |
| HS | SS |  | 0 | 0 |  | 0 |
| SeNu | SS |  | 0 | 0 |  | ND |
| HCMJ136 | SS |  | 0 | 0 |  | ND |
